# Effects of *Hypericum perforatum* extract on IgG titer, leukocytes subset and spleen index in rats

**Published:** 2014

**Authors:** Tahereh Aghili, Javad Arshami, Abdol Mansur Tahmasbi, Ali Reza Haghparast

**Affiliations:** 1 ^*‎*^*Graduate Student in Animal Sciences, College of Agriculture, Ferdowsi University of ‎Mashhad, Mashhad, I. R. Iran*; 2 *‎Department of Animal Sciences, College of Agriculture, Ferdowsi University of ‎Mashhad, Mashhad, I. R. Iran*; 3 *‎Department of Pathology, College of Veterinary Medicine, Ferdowsi of University of ‎Mashhad, Mashhad, I. R. Iran*

**Keywords:** *Hypericum perforatum*, *Immune system*, *Spleen index*, *Rats*

## Abstract

**Objectives:** *Hypericum perforatum L.* is a medicinal plant containing many polyphenolic compounds, ‎including flavonoids and phenolic acids with antidepressant and anti-inflammatory properties. ‎This study was investigated the effects of *Hypericum perforatum* extract (HPE) on immunity, ‎body weight (BW), and spleen index (SI) in rats.‎

**Materials and Methods:** A total of 24 Wistar male rats were randomly received 4 different doses (6 rats each) of HPE ‎‎(0, 100, 200 and 400 mg/kg BW) intraperitoneally for 14 days using a completely ‎randomized design. On days 1 and 7, rats were received 0.5 ml SRBC (10%) injection. Blood ‎samples were collected on day 14 to evaluate IgG titer and leukocyte count. On days 1, 7 and ‎‎14, the BW and on day 14 spleen were weighted for SI. ‎

**Results:** The IgG titer increased with higher doses of HPE. The HPE increased number of ‎lymphocytes at 200 mg but decreased at 400 mg, number of neutrophils decreased at 200 mg ‎but increased at 400 mg, and number of monocytes increased at 100 mg and 200 mg but ‎decreased at 400 mg (p<0.01). Increasing doses of HPE lowered BW (p<0.01). ‎The HPE increased SI at 100 mg and 200 mg but decreased at 400 mg (p>0.072). ‎

**Conclusions:** The results showed that HPE slightly improved IgG titer but significantly increased ‎the number of leukocytes and monocytes at 200 mg, and neutrophils at 400 mg. The HPE ‎decreased BW at 100 mg and 200 mg with no damage on spleen. ‎

## Introduction

‎Today, herbal products are being used increasingly in many countries especially in ‎Iran. In fact, 80% of the world population relies primarily on herbal remedies (Austin, 1998). ‎The herbal products are being used for the enhancement of general health and to boost the ‎immune system ability to combat diseases such as common cold, rheumatoid arthritis, and ‎even cancers (Andrew and Catherine, 1999 ▶). One of the proposed health beneficial herbal ‎medicines is *Hypericum perforatum L*. which is a perennial plant native to Europe and Asia ‎‎(Bombardelli and Morazzoni, 1995 ▶). 

This plant has been used for its medicinal properties in ‎various ways for many years. In the last two decades, the pharmacological studies showed the ‎antidepressant properties of *Hypericum perforatum *(Chatterjee et al., 1998 ▶). Other studies ‎reported that the plant extract has anti-viral (Richer and Davies, 1995 ▶), anti-microbial ‎‎(Fitzpatrick, 1954 ▶), anti-inflammatory (Kumar et al., 2001 ▶), anti-tumor, anti-angiogenic, and ‎anti-oxidant properties (Cuzzocrea et al., 2001 ▶). The main chemical compounds engaged in these ‎activities are naphtodianthrones (hypericins), phloroglucinols (hyperforins) and flavonoids ‎‎(Nahrstedt and Butterweck, 1997 ▶).‎

‎ It has been reported that *Hypericum perforatum* has an immunostimulatory effect *in vitro* ‎(Wilasrusmee et al., 2002 ▶). Another study suggested that the biologically active products ‎obtained from *Hypericum perforatum* could exhibit immunotropic properties (Schempp et al., ‎‎2003). Recently, Jiang et al (2012) ▶ reported that *Hypericum perforatum* extract administration ‎as a dietary supplement in the peri-immunization period improved the humoral immunity of ‎hens to the influenza vaccine. However, Liu et al. (2000) showed that there were significant ‎variations in the amounts of major components among five different brands of *Hypericum ‎perforatum*. In addition, there is a lack of comprehensive studies which determine the certain ‎doses of this plant for human usage and its level of toxicity. Therefore, the aim of present ‎study is to investigate the effects of four different doses of *Hypericum perforatum* extract (0, ‎‎100, 200 and 400 mg/kg body weight) for 14 days on humoral and cellular immunities, body ‎weight and spleen index in Wistar male rats.‎

## Materials and Methods

**Plant material and extract preparation**

Dried plant of *Hypericum perforatum* was prepared from Khorasan Science and Technology Park. The aerial parts of plant were crude and macerated in 50% ethanol then filtered after 72 hrs. The samples were concentrated under reduced pressure in a rotary evaporator at 40°C to remove ethanol. The remaining aqueous part was lyophilized at -80°C (Can-Özgür et al., 2008 ▶). 

**Rats and treatments**

Wistar male rats (200-250g) were obtained from the Pasteur Institute of Tehran, Iran. The rats were maintained in animal house facility in the faculty of Agriculture at Ferdowsi University, Mashhad. The animal house maintained at 22±1ºC with 12hrs light : 12hrs dark cycle. Rats had ad libitum access to standard laboratory chow and tap-water. A total of 24 male rats were randomly assigned into 4 treatment groups of HPE (0, 100, 200 and 400 mg/kg BW) with 6 rats in each treatment group using the completely randomized design. The rats in the treatment groups received HPE hydroalcoholic and the control group received 10 mL normal saline injection (intraperitoneally, i.p.) for 14 days. 

**Humoral antibody response to SRBC**

The SRBCs (sheep red blood cells) test was applied to determine the level of immunoglobulin G (IgG) in different treatment groups. All rats were inoculated with SRBCs (10% suspension in PBS, 0.5 mL of 1×10^8^/μl/rat) on day 1 and challenged on day 7 (Vaibhav and Arun Kumar, 2010, Agrawal et al., 2010). On day 14, blood samples were collected from the heart of rats to determine the concentration of IgG in serum, using a sandwich enzyme linked immune sorbent assay (ELISA) kit (GWB- BAA3A8, Genway-Biotech, USA). 

**Blood leukocyte count**

Blood samples were collected for leukocytes count (%) on day 14, using an optical microscope. For differential of leukocytes, the smear slides were stained with Giemsa then lymphocytes, monocytes, and neutrophils were counted and presented as percentage (Lucas and Jamroz, 1961 ▶).

**Body weight and spleen index**

Body weight of rats was measured on days 1, 7 and 14 in different treatment groups. At the end of experiment, spleen was removed and weighed by an electronic balance and the spleen index was calculated according to the following formula (Xia et al., 2010 ▶):

Spleen index = spleen weight (g)/ body weight (g) ×100 

**Statistical analysis**

The data was analysed by Statistical Analysis System software (9.1) and is shown in mean ± SEM. Statistical analysis was accomplished using one-way analysis of variance (ANOVA) and for post-hoc Duncan’s multiple range test was applied. The significance level was presented at p<0.05.

## Results

**Anti**-**SRBC titers**

The results of immunoglobulin (IgG) responses to different doses of HPE in rats on day 14 are shown in Table 1. The effect of *Hypericum perforatum* hydroalcoholic extract on IgG titers in rats showed no significant differences between treatment groups. The level of IgG titers displays upward trend with increasing HPE doses compared to control group.

**Blood leukocyte count**

The results of leukocyte enumeration (%) on day 14 in rats treated with different levels of HPE are presented in Table 2. The percentage of lymphocytes significantly increased at 200 mg but decreased at 400 mg HPE in rats (p<0.01). The percentage of neutrophils significantly decreased at 200 mg but increased at 400 mg HPE in rats (p<0.01). The percentage of monocytes significantly increased at 100, 200 and 400 mg HPE in rats (p<0.01).

**Body weight**

The results of body weight on days 1, 7, and 14 in rats treated with different doses of HPE are showed in Table 3. The weight of body decreased significantly on days 7 and 14 by increasing HPE doses compared to the control group (p<0.01).

.

**Spleen index**

The results of spleen index in rats treated with different doses of HPE on day 14 are shown in Table 4. The spleen index increased at 100 mg (p<0.05) and 200 mg but decreased at 400 mg HPE in compare to the control group (p<0.072).

**Table 1:** Effects of different doses of *Hypericum perforatum* extract on IgG concentration in male rats

	***Hypericum perforatum*** ** hydroalcoholic extract (mg/kg BW)**			
	**0**	**100**	**200**	**400**
**Level of IgG (ng/ml)**	404.50 ± 21.47	409.61 ± 21.47	414.12 ± 21.47	418.36 ± 21.47

Values are means ± SEM;

* p<0.01, Duncan’s test as compared to the control group.

**Table 2:** Effects of different doses of *Hypericum perforatum* extract on leukocyte subset count in rats

**Parameters (10** ^3^ **/μl)**	***Hypericum perforatum*** ** hydroalcoholic extract (mg/kg BW)**			
	**0**	**100**	**200**	**400**
**Lymphocytes**	67.00 ± 3.14	65.66 ± 3.14	74.66 ± 3.14*	49.33 ± 3.14*
**Neutrophils**	27.66 ± 1.15	25.66 ± 1.15	19.65 ± 1.15*	43.00 ± 1.15*
**Monocytes**	5.30 ± 0.86	15.00 ± 0.86*	8.66 ± 0.86*	10.66 ± 0.86*

Values are means ± SEM;

* p<0.01, Duncan’s test as compared to the control group.

**Table 3:** Effects of *Hypericum perforatum* extract on body weight in days 1, 7 and 14 in male rats

**Body weight (g)**	***Hypericum perforatum*** ** hydroalcoholic extract (mg/kg BW)**			
	**0**	**100**	**200**	**400**
**1st day**	213	231	214	210
**7th day**	251± 6.10	218 ± 6.10*	211 ± 6.10*	198 ± 6.10*
**14th day**	258 ± 5.67	225 ± 5.67*	219 ± 5.67*	209 ± 5.67*

Values are means ± SEM;

* p<0.01, Duncan’s test as compared to the control group.

**Table 4:** Effects of different levels of *Hypericum perforatum* extract on spleen index in male rats

**Parameter**	***Hypericum perforatum*** ** hydroalcoholic extract (mg/kg BW)**			
	**0**	**100**	**200**	**400**
**Spleen Index (mg)**	3.70 ± 0.30	4.70 ± 0.30*	4.20 ± 0.30	3.40 ± 0.30

Values are means ± SEM;

* p<0.05, Duncan’s test as compared to the control group.

## Discussion

Most herbal medicines contain chemicals such as hypericins, hyperforins, and flavonoids that could affect the immune system in different ways (Wilasrusmee et al., 2002 ▶; Jiang et al., 2012 ▶). Some studies have reported that *Hypericum perforatum* had significant therapeutic efficacy and could improve immunologic functions for chickens infected experimentally with IBDV and AIV, respectively (Ruofeng et al., 2012; Landy et al., 2012 ▶). In a recent study, researchers showed that orally-administered *Hypericum perforatum* extract (HPE) could stimulate the SOCS3 pathway (transcription of suppressor of cytokine signalling 3) and consequently cause impaired immune defense against influenza virus infection which could lead to higher mortality in mice (Huang et al., 2013 ▶). In this experiment, although the IgG concentrations in rats injected with different levels of HPE was not significant, however as the doses of HPE increased, the IgG titer increased as well. Likewise, oral administrations of HPE to mice infected with the influenza A virus (H1N1) was highly effective in preventing of death (Xiuying et al., 2009). In addition, another study reported immunostimulating activity of polyphenol fraction of *Hypericum perforatum *with respect to the system of mononuclear phagocyte system, cellular and humoral immunity (Evstifeeva and Sibiriak, 1996 ▶). 

Lymphocytes are a type of white blood cells that provide a means for humoral and cellular immunities and usually increases in the presence of infection or antigens. In this study, the number of lymphocytes differ significantly among doses, though it tended to increase at 200 mg and decrease at 400 mg HPE (p<0.01). Similarly, in other trials in mice, the use of *Hypericum scabrum *extract had significant effect on lymphocytes, neutrophils, and eosinophils that increased in treatment group (Pirbaluti et al., 2011 ▶). In this research, the number of neutrophils decreased at 200 mg but increased at 400 mg and the number of monocytes increased in all treatments (P<0.01). However, it has been suggested that high dose of external substance can lead to consequences of stress in body which results in demargination of neutrophils (Bafor and Igbinoun, 2008 ▶). Overall, this study showed that HPE at 200 mg/kg BW is a safe dose to be used and has beneficial effects on immune system of rats.

The results obtained from this study indicated that HPE treatments reduced body weight in rats within 14 days. In contrast, weight gain has reported in mice treated with 100 and 1000 mg/kg BW of *Hypericum scabrum *extract for 2 weeks (Pirbaluti et al., 2011 ▶). Several studies suggested that HPE increases monoamine levels in synaptic clefts (Hirano et al., 2004 ▶; Müller, 2003b ▶). Furthermore, it is suggested that the hypophagic effect of HPE seems to be related to the rise in monoamine levels in central nervous system of rats (Can Özgür et al., 2008 ▶). It also has been reported that activation of some serotonergic receptor subtypes (5-HT2C and 5-HT1B) reduces appetite and food intake (Dalton et al., 2006 ▶; Bickerdike, 2003 ▶). In the present study, a decreasing trend of body weight with increasing doses during 7 and 14 days was observed.

Spleen functionally filters red blood cells and involves in active immune response through humoral and cell-mediated pathways. It has been reported that the spleen contains in its reserve half of the body’s monocytes within the red pulp (Weissleder and Pittet, 2009 ▶). In this experiment, the HPE increased spleen index at 100 mg and 200 mg which represent improvement of spleen activity, however, the SI decreased at 400 mg. So, it is possible that the value of 400 mg HPE might cause toxicity (functional asplenia). 

The result of this study provides new evidences about the mechanism of action of *Hypericum perforatum L.* which is a dose-dependent influence on the immune system. The results suggested that the safe and effective level of HPE is 200 mg/kg BW and the 400 mg may cause toxicity in spleen and reduce body weight in rats. 
